# Improving palliative care for people with intellectual disability: a self-assessment of policies, practices and competencies in care services

**DOI:** 10.1186/s12904-023-01224-2

**Published:** 2023-07-22

**Authors:** Hille Voss, Anneke L. Francke, Anke J.E. de Veer

**Affiliations:** 1 NIVEL, Netherlands Institute for Health Services Research, P.O. Box 1568, Utrecht, 3500 BN The Netherlands; 2 Amsterdam UMC location Vrije Universiteit Amsterdam, APH Amsterdam Public Health research institute, Van der Boechorststraat 7, Amsterdam, 1081 BT The Netherlands

**Keywords:** Palliative care, End-of-life, Intellectual disability, Care services, Competencies, Policy, Training

## Abstract

**Background:**

Providing care for ageing and vulnerable people with intellectual disability (ID) is challenging, and professionals working in ID care often have limited experience in palliative care. The current study provides insight into palliative care practices in ID care services and competencies of professionals and identifies ways to improve palliative care for people with ID.

**Methods:**

For this study ten services in the Netherlands were recruited that provide care for people with mild to profound ID. Professionals in each of these services conducted a self-assessment of their palliative care policies and practices based on nine core element of palliative care described in the Dutch Quality Framework for Palliative Care. The self-assessment included a medical file review of a total of 100 people with ID who died non-suddenly. In addition, 424 professionals from the services returned a digital questionnaire on palliative care competencies and training needs.

**Results:**

The self-assessments showed that individual care plans were recorded for people with ID and that multidisciplinary teams provided physical, psychological, social and spiritual care. However, other core elements of palliative care, such as cooperation with other organisations and expertise in palliative care, were less present in ID care services. Only half of the services collaborated with regional organisations in palliative care, and most services listed no requirements for the palliative care skills of their professionals. The questionnaire showed that almost 10% of the professionals reported that they were not at all competent in providing palliative care, and 74% felt that they needed training in palliative care. Reported areas for improvement in the provision of palliative care were increasing the quality of palliative care, improving the expertise of professionals and identifying palliative care needs earlier.

**Conclusions:**

To improve palliative care in ID care services changes are required both in competencies of professionals, and organisational policies and practices. Services should enhance awareness about palliative care for people with ID, strengthen collaboration with palliative care services, and offer training or support for professionals in assessing and meeting the needs of people with ID at the end of life.

**Supplementary Information:**

The online version contains supplementary material available at 10.1186/s12904-023-01224-2.

## Introduction

People with intellectual disability (ID) comprise approximately 1–1.5% of the global population [1]. They have a significantly reduced ability to understand new or complex information and to learn and apply new skills [2, 3]. Due to improvements in health and social care in recent decades, the life expectancy of people with ID in Western countries has increased [4]. As a result, a growing number of people with ID experience life-threatening health problems when they age, such as progressive respiratory and circulatory diseases, cancer and dementia [5, 6]. On the other hand, many people with severe or profound ID may also have physical health issues that present at birth or shortly thereafter, such as epilepsy, pulmonary health problems and reflux disease [7]. Care services and ID professionals should therefore be prepared to provide palliative care for this growing population of ageing and vulnerable individuals.

Providing palliative care for people with ID requires a shift in the care approach [8]. Instead of encouraging individuals to undertake activities and acquire new skills, palliative care focusses on providing comfort and symptom management [8–10]. In the Netherlands, professionals who provide daily care or support for people with ID were often trained initially as social workers. Consequently, they received no training in palliative care and many feel inadequately equipped to provide this care [11].

In addition, previous research has shown that these professionals do not know how to inform people with ID about dying and death and tend to avoid discussions about the end of life. This is because they believe people with ID may be unable to cope with such discussions or because professionals want to protect themselves from possible distress [12–14]. As a result, many people with ID have limited understanding of dying and death and are often not told that they are dying [15, 16]. However, also for people with ID, it is important to know and plan for their own death [17].

A systematic review of the palliative care needs of people with ID showed that their needs at the end of life are equivalent to those of the general population and include physical, psychological, social and spiritual aspects [10]. A challenge is that, because of difficulties in communication, assessing, understanding and addressing the needs of people with ID at the end of life is often complex [16, 18, 19]. In addition, in settings such as nursing homes and hospices, palliative care is provided structurally, while in some ID care settings, professionals may not be called on to provide palliative care or deal with the death of an individual for a number of years [20]. Therefore, professionals working in ID care often have little or no experience in providing palliative care.

To support professionals, a growing number of innovations have become available in recent decades to improve the provision of palliative care in various care settings [21]. A recent study showed that innovations that were originally developed for the general population of palliative care patients can also be successfully implemented in services for people with ID, but require adaption to the specific care setting [22]. To attain sustainable improvement in palliative care, it is therefore important to assess (a) whether the care innovation is compatible with existing practices within the organisation and (b) whether the innovation is in line with values of the professionals concerned [23].

The present study provides insight into palliative care policies and practices as well as into the competencies and training needs of professionals in ID care services. Taking these aspects into account, the overall aim of this study was to identify needs for improvement in the provision of palliative care for people with ID. We therefore addressed the following research questions: (1) What are the current palliative care policies and practices in ID care services in the Netherlands?, (2) How do the professionals involved perceive their competencies for providing palliative care?, and (3) What improvements in palliative care are needed according to these professionals?

## Method

### Setting

This study was part of a three-year project focusing on implementing innovations to improve palliative care in Dutch ID care. The project was funded by the Netherlands Organization for Health Research and Development (ZonMw). Initially, 11 ID care services were involved in the project, but one ID care service withdrew from the project due to COVID-19. The ID care services varied in size, and provided care to approximately 450 to a maximum of 6,000 (median 1,375) people with mild-to-severe or profound ID living in group homes in the community and/or in residential settings. Of the ten remaining services, seven also provided care for people with other or additional disabilities including sensory disability, physical disability, acquired brain injury, chronic and progressive disease, psychiatric disability or autism disorder.

### Design and data collection

Data was collected at the start of each implementation project, between September 2020 and December 2021. Data collection included (1) a self-assessment of palliative care policies and practices within each ID care service and (2) a digital questionnaire distributed to professionals employed at the ID care services.

Firstly, the self-assessment was based on an existing Dutch digital tool [24] and consisted of a medical file review of the last ten patients who died non-suddenly within a care organisation, and a questionnaire about nine core elements of palliative care. These core elements consisted of (1) timely identification of palliative care needs, (2) shared decision-making, (3) advance care planning, (4) attention for the various dimensions of palliative care (physical, psychological, social, and spiritual), (5) palliative care expertise, (6) coordination and continuity of care, (7) cooperation with other organisations, (8) individual care plans and (9) support for professionals. For each element a standard had previously been formulated and approved in the Quality Framework for Palliative Care in the Netherlands [25], see Table 1.

**Table 1:** Nine core elements and associated standards of palliative care according to the Netherlands Quality Framework for Palliative Care [25]

Core elements of palliative care	Standard
1. Identification	Patients in need of palliative care are identified in a timely matter.
2. Shared decision-making	Shared decision making is incorporated in palliative care as a continuous process in which care is tailored to the individual’s situation and to the achievable values, wishes and needs of individual patients and their relatives.
3. Advance care planning	Advance care planning is addressed in a timely manner and decisions are recorded in the individual care plan. When the situation requires this, plans will be adjusted.
4. Dimensions of palliative care - Physical - Psychological - Social - Spiritual	Physical: The physical symptoms of a patient with a life-threatening condition or vulnerability are treated in accordance with applicable guidelines, which may be deviated from on the basis of knowledge and expertise, and treatment is tailored to individual patients. Medical instruments and resources must be available in sufficient quantities. Psychological: Together with the patient and their relatives, the care provider pays attention to the psychological consequences of a life-threatening condition or vulnerability and the presence of any psychiatric symptoms. Social: The care provider, together with the patient and their relatives, considers their social context, in order to meet their goals, wishes and needs. In this way, strengths can be used and the well-being of the patient and their relatives can be enhanced. Spiritual: Attention is focused on what is meaningful for the patient and their relatives and appropriate support is provided for spiritual and existential questions and needs.
5. Expertise	Professionals are qualified for the care they provide and keep their knowledge up to date with relevant training.
6. Coordination and continuity	A personal and dynamic team of professionals is formed around the patient and their relatives, who are available at all times. This team works on the basis of the individual care plan, with the central care provider as the connecting link.
7. Cooperation with other organisations	Relevant organisations within the same region work together effectively and efficiently to meet the wishes and needs of patients with a life-threatening condition or vulnerability and the wishes and needs of their relatives.
8. Individual care plan	Each patient in the palliative phase has an individual care plan that accompanies the patient and is adjusted if necessary during the disease process. The individual care plan includes agreements that are readily accessible, also during nights, weekends, in crisis situations and in the dying phase.
9. Support for professionals	Professionals are aware of the emotional impact of providing palliative care. They reflect on their own attitude and actions and have an eye for their personal balance.

The self-assessment included open and closed questions about whether palliative care is provided according to the standard. These questions could be answered using the medical file reviews and/or policy documents of the organisation. For example, two questions regarding the second core element - shared decision-making - included: “In how many of the files of the last 10 non-unexpectedly deceased patients is something recorded about shared decision-making?”, and “How and where are the agreements arising from the shared decision-making process in the file recorded?”. At each ID care service, the self-assessment was done by a group of professionals from various disciplines (social workers, physicians, nurses and other professionals) as well as managers of the ID care services. The assessment groups consisted of two to five individuals. A digital report was automatically generated after completing the questions of the self-assessment.

Secondly, a self-developed questionnaire was sent by email to professionals at the ten participating ID care services. The questionnaire was based on questions from a previously developed questionnaire used in another study on experiences with palliative care that were found to be reliable and valid [26]. To test the questionnaire for use in the current study, we conducted a cognitive interview with a nurse at an ID care service that did not participate in this study. Based on the interview, we reformulated some questions (e.g. “instruments” was changed to “tools”) and added an explanation of the Dutch Quality Framework for Palliative Care [25]. The questionnaire included closed questions about palliative care practices at the ID service, the perceived quality of palliative care and the professionals’ self-perceived competencies in providing palliative care. In addition, an open-ended question was included to enable the professionals to explain the necessary improvements in palliative care. Questions on background characteristics (sex, age, profession, years of experience, average working hours per week and previous training in palliative care) were included at the end of the questionnaire. The survey questions used in this manuscript are included in a supplementary file.

### Analyses

An extraction form was developed by two researchers (HV and AdV) that was used to extract and summarise the results of the self-assessments and the extent to which the nine palliative care standards were met at the ten participating ID care services. The extraction of the self-assessments was conducted independently by two researchers (HV and AdV). The completed extraction forms were processed in Excel. Extracted information was compared until agreement was reached, and continuously discussed to formulate key insights regarding the nine palliative care standards. Results from the digital questionnaire were analysed using Stata version 15. To test differences between groups, Pearson’s chi-square tests were used.

## Results

### Self-assessments of palliative care

The results of the self-assessments including the medical file review of non-suddenly deceased people with ID (n = 100) showed that palliative care in the ten ID care services was not being provided entirely according to the standards of the Netherlands Quality Framework for Palliative Care [25]. The results of the self-assessments for each core element of palliative care are described in more detail below.

#### Identification

To identify people with ID in need of palliative care, professionals in nine out of the ten participating ID care services used the ‘surprise question’: “Would you be surprised if the patient died within the next year?”. If the answer is no, the patient can be identified as “in need for palliative care”. However, in most ID care services it remained unclear when or by whom the surprise question is answered or how palliative care needs were identified:﻿

“The question: ‘is a patient still doing well, or are they at the terminal stage?’ is asked when a patient feels poorly and when there are concerns. These concerns are shared with colleagues and the patient’s family. If necessary, the GP and the behavioural expert are called in. (…) These actions are being initiated, but no clear transition is made with regard to the palliative phase. Switching is more frequent when the terminal phase presents itself.” – ID care service no. 7.

#### Shared decision-making

Shared decision-making in all participating ID care services took place in regular multidisciplinary team meetings in which the care plan of the individual with ID is discussed. These meetings were held with the professionals involved, relatives and, if possible, the person with ID. At most of the ID care services, decisions were recorded in the individual care plans. One service used a separate ‘palliative file’ for people with ID who receive palliative care. If necessary, decisions regarding people with ID in the palliative phase were reviewed more frequently and changed if necessary:

“In any case, an evaluation of the care plan takes place once a year with the personal supervisor [daily care professional], a relative and, if possible, the client is also present. Decisions are recorded in the electronic patient file, in the evaluation of the care plan or as separate reports of conversations and agreements. If necessary, there is more frequent contact about decisions, progress, goals and expectations.” – ID care service no. 5.

#### Advance care planning (ACP)

Eight of the ten services mentioned that ACP conversations with people with ID and/or relatives took place. Some ID care services used a workbook on end-of-life care for people with ID to discuss wishes and needs for future care. ACP discussions with relatives focused on quality of life and medical end-of-life decisions:

“ACP discussions often take place with legal representatives. The main focus is on comfort and quality of life. Decisions are often made about non-resuscitation, whether or not to be sent to hospital or IC, and decisions regarding COVID-19 are also being made at present.”– ID care service no. 1.

#### Dimensions of palliative care

Multidisciplinary teams including various professionals were involved in providing physical, psychological, social, and spiritual care. At all participating ID care services, a physician specializing in care for people with ID provided physical care. In addition, social workers and behavioural experts were involved in psychological and social care. Eight of ten services employed a spiritual counselor to provide spiritual care for people with ID in the palliative phase:

“Support is given by the spiritual counselor at the request of the team that supports the person. In addition, as part of our care we counsel clients closely and in this way we also try to bring the psychological dimension up for discussion. The behavioural expert also has a role in this phase of life.” – ID care service no. 6.

#### Expertise

There were limited training opportunities in palliative care for professionals in the participating ID care services: only one service offered standard training in palliative care for all professionals. To disseminate knowledge, nurses specializing in palliative care were employed at six of the ten services as palliative care consultants. Eight of the ten ID care services listed no requirements on the necessary skills and specific expertise in palliative care of ID care professionals:

“Primary care is aimed at the well-being of the patient in various respects. Different care requirements have been established for the various patient groups. To my knowledge, there are no requirements for palliative care.” – ID care service no. 8.

#### Coordination and continuity

In all but one of the participating ID care services, care for individuals with ID was coordinated by a professional – often a social worker or nurse – involved in the daily care of the individual with ID. This professional also had a central role in the coordination of palliative care:

“Every resident has a coordinating daily care professional who acts as a case manager and in that role is the central figure for the patient, their representatives and for employees. This professional is responsible for implementing the individual care plans of patients in a residential facility and is therefore also the primary contact person.” – ID care service no. 4.

#### Cooperation with other organisations

All participating ID care services were part of a regional palliative care network, which is a formal and sustainable partnership of independent organisations (e.g. nursing homes, psychiatry, hospitals, hospices and community care) that are involved in palliative care in a specific region. However, collaboration within the palliative care network was often incidental. Only half of the services made agreements or formulated protocols with other organisations in palliative care:

“[Name ID care service] is part of the regional palliative care network. There are no binding agreements with regard to palliative care within the network for collaboration regarding the patient, or transfer and referral procedures.” – ID care service no. 10.

#### Individual care plan

All participating ID care services had an individual care plan for the last ten patients who died non-suddenly. Individual care plans were regularly reviewed in the multidisciplinary team meetings. It was not always explicitly stated in the care plan that a client was in the palliative phase. The individual care plans did, however, report on medical end-of-life decisions, such as a resuscitation policy or wishes for the admission to the hospital:

“Medical policy agreements are recorded in the care plan; they are sometimes reconsidered in the palliative phase and sometimes again in the palliative terminal phase.” – ID care service no. 6.

#### Support for professionals

Nine of the ten ID care services had a formal policy on ‘support for professionals’ to enable reflection on their attitude and the impact of providing palliative care, for example by providing intervision sessions for peers and supervisors. At all of the ID care services, moral deliberation sessions were offered to enable professionals to discuss personal or professional dilemmas or issues and to express emotions:

“’Care for the carers’ is offered and encouraged in our organisation. There are also opportunities for intervision and supervision for professionals. A meeting with the spiritual caregiver and/or a confidential advisor is also possible. The intention is that every team has a moral deliberation session at least once a year.” – ID care service no. 4.

### Questionnaire on palliative care

The questionnaire was returned by 424 professionals from the ten participating ID care services (response rate 64%), of whom 299 filled in the questionnaire completely. This involved between 14 and 49 professionals at each ID care service. Of the respondents, 58% (n = 223) had supported at least one person with ID in need for palliative care during the past year. Professionals who fully completed the questionnaire reported more often that they were involved in the provision of palliative care for a person with ID during the past year than professionals who did not fully complete the questionnaire (X^2^ [2] = 13.23, p < 0.001).

Background characteristics of participants are listed in Table 2. Most respondents were female (90%), and provided daily care for people with ID (68%). Of the professionals who provided daily care (n = 222), 52% was trained as a social worker and 48% as a nurse. Professionals reported working an average of 26 h per week (minimum 4 h; maximum 40 h). The majority (72%) reported that they had not received previous training in palliative care, and 74% reported a need for training (or more training) in palliative care. Participants who reported receiving previous training indicated that they had attended conferences or workshops on palliative care or had participated in training focusing on ageing people with ID and providing grief support for people with ID.

**Table 2:** Background characteristics of professionals

	n	%
*Age (n = 298)*		
< 30 years	65	22
30–49 years	115	38
≥ 50 years	118	40
*Sex (n = 292)*		
Female	264	90
Male	25	9
Prefer not to say	3	1
Profession (n = 328)*		
Daily care staff	222	68
*Social worker (52%)*		
*Nurse (48%)*		
Nursing staff	29	8
Manager	20	6
Behavioural expert (e.g. psychologist)	12	4
Allied health professional (e.g. physiotherapist, speech therapist, dietician)	6	2
Physician	5	2
*Specialised physician for people with ID (n = 2)*		
*General practitioner (n = 3)*		
Spiritual counselor (pastor)	4	1
Palliative care specialist	3	1
Other (e.g. student, assistant, night care worker, trainer, musical therapist)	27	8
*Work experience in ID care (n = 296)*		
< 1 year	12	4
1–5 years	81	28
5–10 years	41	14
10–20 years	78	27
> 20 years	82	28
*Previous training in palliative care (n = 296)*		
No	214	72
Yes	82	28
*Need for training (or more training) in palliative care (n = 295)*		
No	77	26
Yes	218	74

*categories are not mutually exclusive

#### Perceived competencies of professionals

Of the participants (n = 302), 9% reported feeling “not competent at all” in providing palliative care, 33% reported feeling “somewhat competent”, 43% reported feeling “reasonably competent” and 15% of professionals reported feeling “largely competent”. The professionals who reported most often that they felt “largely competent” in providing palliative care were palliative care specialists (100%), spiritual counselors (50%), nursing staff (41%), and physicians (40%); none of these professionals reported feeling “not competent at all”. Daily care staff with a background in nursing more often reported feeling “largely competent” (22%) compared to daily care professionals with a background in social work (7%) (p < 0.01).

Table 3 lists specific competencies in palliative care. Participants felt most competent (> 30% “definitely skilled”) in providing physical care, reporting palliative care needs, evaluating provided care, providing care during the last days of life and reflecting on their own attitudes and behaviour. Participants felt least competent (> 15% “probably not skilled”) in proactively discussing wishes and needs for future care with people with ID, discussing moral dilemmas, planning and organising palliative care, and collaborating with other organisations specialised in palliative care.

**Table 3:** Competencies in palliative care reported by professionals

	n	Definitely skilled	Likely skilled	Probably skilled	Probably not skilled
Identifying palliative care needs	301	11%	35%	46%	8%
Systematically identifying symptoms, problems and needs	299	13%	31%	43%	13%
Proactively discussing wishes and needs for future care with people with ID	299	13%	28%	43%	16%
Proactively discussing wishes and needs for future care with relatives	302	25%	32%	33%	10%
Involving person with ID and relatives in decisions in palliative care	300	27%	33%	34%	6%
Discussing moral dilemmas with people with ID and/or relatives	301	17%	29%	38%	16%
Providing physical care	301	33%	31%	28%	9%
Dealing with psychological symptoms and problems	301	20%	42%	30%	8%
Supporting the social wellbeing of people with ID	300	22%	43%	29%	5%
Supporting the spiritual wellbeing of people with ID	302	12%	33%	39%	15%
Reporting symptoms, wishes and needs of people with ID	302	38%	38%	20%	4%
Actively involving relatives in care for people with ID	299	29%	34%	28%	9%
Collaborating with other professionals in palliative care	300	28%	37%	28%	7%
Planning and organising palliative care	303	18%	31%	33%	18%
Collaborating with care organisations specialised in palliative care	300	9%	28%	39%	24%
Evaluating the provided palliative care	300	32%	38%	25%	5%
Promoting the importance of palliative care within the organisation	302	23%	38%	32%	7%
Supporting relatives in their grief	298	17%	38%	35%	10%
Supporting people with ID in their grief	301	29%	42%	24%	5%
Supporting professionals who are having a difficult time	302	30%	37%	28%	5%
Providing care during the last days of life of a person with ID	302	39%	36%	20%	5%
Reflecting on own attitudes and behaviour	302	33%	42%	22%	3%

#### Need for improvement in palliative care

Table 4 shows the needed improvements in palliative care reported by professionals. The top 3 needed improvements were (1) enhancing the expertise of the professionals involved in palliative care, (2) identifying palliative care needs, and (3) improving the quality of palliative care for people with ID and their relatives. These needed improvements were reported by 52%, 42%, and 33% of professionals, respectively. Enhancing the expertise in palliative care was needed according to professionals because team members who provided daily care for the person with ID had limited expertise and experience in this area: “We have a young team, with little experience in palliative care. More knowledge is important to enhance the expertise of the team. Due to this lack of experience, the concept of palliative care is often unclear and the necessity for such care is not properly identified.” - ID care service no. 10.

**Table 4:** Needed improvements in palliative care reported by professionals (n = 317)

Enhancing the expertise of professionals involved in palliative care	52%
Identifying palliative care needs	42%
Improving the quality of palliative care for people with ID and their relatives	33%
Advance care planning	21%
Collaborating with other professionals in palliative care	21%
Planning and organising palliative care	19%
Collaborating with other care organisations specialised in palliative care	17%
Documenting wishes and decisions (and corresponding changes) in the individual care plan	8%
Supporting relatives and involving them in palliative care for their loved one	7%
Involving people with ID and relatives in decision-making	5%
I don’t know	9%
Other*	6%
None of the above	4%

*e.g. dealing with grief, providing care during the night, reflecting on palliative care provision, solving staffing issues, having sufficient medical devices available

Professionals explained the importance of improving the identification of palliative care needs, including recognizing that the person with ID is in the palliative phase: “This is because timely identification in the organisation does not always happen, but it is important for high-quality palliative care. I think there are many patients who actually receive palliative care, but that concept is not yet used deliberately (nor is it included in the care plan). If the need for such care is identified promptly, we can also involve relatives more effectively and sooner in the process and thus support them better. This also improves the quality of palliative care. At an early stage it is also important to discuss the wishes for future treatments and care with all those involved. That deserves attention.” - ID care service no. 6.

Improving the quality of palliative care for people with ID and their relatives was reported by professionals as needing improvement in relation to the expertise of professionals in palliative care:

“There is still room for improving the quality of care. It would be nice if employees could specialize in this, in teams where the expectation of palliative care is highest, e.g. aging patients. (…) Although I think that every employee should receive training in the basic principles of palliative care.” - ID care service no. 6.

## Discussion

The present study provides insight into policies, practices and competencies of professionals in palliative care for people with ID. Strengths of ID care services in the Netherlands include the provision of person-centered and multidisciplinary care, which is important to provide high-quality palliative care [18, 27]. At all participating ID care services, individual care plans were recorded and regularly evaluated for every person with ID. In addition, various professionals were involved in providing physical, psychological, social and spiritual support for people with ID, including social workers, nursing staff, specialised physicians for people with ID, behavioural experts, spiritual counselors and, at some ID care services, specialist palliative care staff. This multidisciplinary approach might be unique to the Netherlands, as healthcare policies and practices in Dutch ID care services stipulate the involvement of professionals specialised in providing care for people with disabilities [28].

However, other aspects also require improvement: policies and practices do not focus on timely identification of palliative care needs and ACP is not embedded in the participating ID care services. Collaboration with organisations specialised in palliative care is often absent, and little training is available for ID professionals, which was also highlighted in previous studies [9, 10, 16, 29]. Many ID care professionals at the participating services, especially social workers, reported a need for training in palliative care and felt insecure about their competencies in identifying symptoms, problems and needs, supporting the spiritual wellbeing of people with ID, and planning and organising palliative care.

Developing and offering training opportunities for professionals in identifying, assessing and understanding palliative care needs is especially important in this sector. Due to the various and unique support needs of people with ID, recognizing and communicating about needs, wishes and preferences in palliative care is often difficult [17, 28, 29]. People with ID have their own unique, preferred ways of communicating, and information should be available in accessible formats such as augmentative communication systems, signs or pictorial formats [16, 17]. Professionals in ID care should be trained in identifying symptoms that indicate a need for palliative care and proactively discussing wishes for future care (ACP) tailored to the person’s needs and preferences to support quality end-of-life care for people with ID [13, 17].

In addition to training, ID care services should ensure that adequate support is available for professionals providing care for people with ID in the palliative phase [16]. This could include advisory support of professionals specialised in palliative care who can then disseminate their knowledge among other professionals within the service. Also needed is the development of organisational policies on how to recognize palliative care needs and when to start ACP. These policies can guide professionals and inform them about who has expertise in this area within the organisation. In addition, managers and professionals should enhance awareness about the importance of high-quality palliative care for people with ID and should promote and build partnerships with specialist palliative care services, such as hospices, to share knowledge and skills, and to assess and meet the holistic needs of people with ID at the end of life [30, 31].

Several strengths and limitations of our study should be noted. An important strength is that we examined not only the policies and practices of the ID care services, but also the competencies and experiences of professionals. This enables attainment of systemic and sustainable change in palliative care [22, 29]. Ten ID care services throughout the Netherlands took part in this study, a total of 100 medical files of non-suddenly deceased patients with ID were studied, and 424 professionals filled in the questionnaire, which provided a comprehensive overview of organisational practices and competencies of professionals.

One limitation of this study concerns a possible participation bias concerning the ID care services. This study is a first part of a larger project aimed at improving palliative care for people with ID. It therefore focused on services whose staff could have a relatively large interest in providing palliative care and may therefore not be a representative sample of services in all regards. A second limitation is that there was a considerable amount of missing data in our study; 299 of the 424 professionals filled in all the questions. Professionals who provided palliative care during the past year were more likely to complete the questionnaire compared to professionals who did not provide palliative care, which might indicate that questions were too specific for professionals who did not have much experience with palliative care.

## Conclusions

Several core elements of palliative care, such as individual care plans and multidisciplinary care teams, were present in the policies and practices of the participating ID care services. However, other core elements such as cooperation with other organisations and expertise in palliative care were not present. To attain sustainable improvement in palliative care for people with ID, organisational policies and practices and the competencies of professionals all require attention and improvement. Regarding organisational policies and practices, improvements should focus on enhancing awareness of the importance of palliative care for people with ID, strengthening structural collaboration with other services specialised in palliative care, and offering training or support for professionals in providing palliative care. Regarding individual competencies of professionals, improvements should focus on identifying palliative care symptoms and ACP tailored to individual needs and preferences.

## Electronic supplementary material

Below is the link to the electronic supplementary material.

Supplementary Material 1

## Data Availability

The datasets used and/or analysed during the current study are available from the corresponding author on reasonable request.

## List of Abbreviations

ACP: Advance Care Planning
ID: Intellectual disability
