# Supplementary material for: Improving palliative care for people with intellectual disability: a self-assessment of policies, practices and competencies in care services
Source: BMC Palliat Care. 2023 Jul 22;22:103. doi: 10.1186/s12904-023-01224-2 (PMC10362573; doi:10.1186/s12904-023-01224-2)
Supplement: Supplementary file 1 — Supplementary Material 1 [file 12904_2023_1224_MOESM1_ESM.docx]

Supplementary material SURVEY QUESTIONS (English translation)

This questionnaire was originally presented in Dutch. This document contains the questions on areas of needs for improvement, competencies, and background characteristics.

| Quality of palliative care |
| --- |

Which of the listed conditions need to be improved? *(you can tick a maximum of 3)*

- Documenting wishes and decisions (and corresponding changes) in the individual care plan
- Identifying palliative care needs
- Involving people with ID and relatives in decision-making
- Collaborating with other care organisations specialised in palliative care
- Advance care planning
- Collaborating with other professionals in palliative care
- Enhancing the expertise of professionals involved in palliative care
- Improving the quality of palliative care for people with ID and their relatives
- Supporting relatives and involving them in palliative care for their loved one
- Planning and organising palliative care
- Other, namely ______________________________________________________
- None of the above
- I don’t know

Can you explain your answer? Why do you mention these conditions in particular?

(open ended question)

| **Competencies** |
| --- |

1. To what extent do you feel competent in providing palliative care?

- - Not competent at all
  - Somewhat competent
  - Reasonably competent
  - Largely competent
    - 1. To what extent are you able to perform each skill?

|  | Probably not skilled | | | Probably skilled | | Likely skilled | | Definitely skilled |
| --- | --- | --- | --- | --- | --- | --- | --- | --- |
| 1. Identifying palliative care needs |  | | |  | |  | |  |
| 1. Systematically identifying symptoms, problems and needs |  | | |  | |  | |  |
| 1. Proactively discussing wishes and needs for future care with people with ID | |  |  | |  | |  | |
| 1. Proactively discussing wishes and needs for future care with relatives | |  |  | |  | |  | |
| 1. Involving person with ID and relatives in decisions in palliative care |  | | |  | |  | |  |

| 1. Discussing moral dilemmas with people with ID and/or relatives |  | | |  | |  | |  |
| --- | --- | --- | --- | --- | --- | --- | --- | --- |
| 1. Providing physical care (physical problems or symptoms such as pain, constipation, nausea, shortness of breath) |  | | |  | |  | |  |
| 1. Dealing with psychological symptoms and problems (such as anxiety, depression) |  | | |  | |  | |  |
| 1. Supporting the social wellbeing of people with ID (such as social contacts, daily routines) |  | | |  | |  | |  |
| 1. Supporting the spiritual wellbeing of people with ID (such as dealing with illness and vulnerability, death and dying, beliefs) |  | | |  | |  | |  |
| 1. Reporting symptoms, wishes and needs of people with ID | |  |  | |  | |  | |
| 1. Actively involving relatives in care for people with ID (such as giving information, support before and after death) | |  |  | |  | |  | |
| 1. Collaborating with other professionals in palliative care | |  |  | |  | |  | |
| 1. Planning and organising palliative care (such as ensuring a proper division of tasks, making use of available facilities and knowledge) | |  |  | |  | |  | |
| 1. Collaborating with care organisations specialised in palliative care in the region | |  |  | |  | |  | |
| 1. Evaluating the provided palliative care together with colleagues | |  |  | |  | |  | |
| 1. Promoting the importance of palliative care within the organisation | |  |  | |  | |  | |
| 1. Supporting relatives in their grief | |  |  | |  | |  | |
| 1. Supporting people with ID in their grief (such as fellow residents) | |  |  | |  | |  | |
| 1. Supporting professionals who are having a difficult time | |  |  | |  | |  | |
| 1. Providing care during the last days of life of a person with ID | |  |  | |  | |  | |
| 1. Reflecting on own attitudes and behaviour | |  |  | |  | |  | |

| **Background characteristics** |
| --- |

Finally, we would like to ask you to fill in some background information about yourself.

What is your gender?

- Male
- Female
- Prefer not to say

1. What is your age?

- Younger than 20 years
- 20 – 29 years
- 30 – 39 years
- 40 – 49 years
- 50 – 59 years
- 60 years or more

1. What is your profession in <ID care service>? *(You can tick multiple answers)*

- Social worker 🡪 go to question 4
- Nurse 🡪 go to question 6
- Palliative care specialist 🡪 go to question 6
- team leader, coordinator 🡪 go to question 6
- Behavional expert (e.g. psychologist) 🡪 go to question 6
- Physician 🡪 go to question 5
- Spiritual counselor 🡪 go to question 6
- Allied health professional (e.g. physiotherapist, speech therapist, dietician), namely _____________________ 🡪 go to question 6
- Other, namely_______________________🡪 go to question 6

1. Are you trained as a registered nurse or certified nursing assistant?

- No
- Yes, a registered nurse
- Yes, Ja, certified nursing assistant

🡪 go to question 6

1. What training have you had?

- Specialized physician for people with ID
- General Pracitioner
- Physician (basic training)
- Other, namely____________________

1. How many years have you worked in the care of people with ID?

- Less than 1 year
- 1 - 5 years
- 5 - 10 years
- 10 - 20 years
- 20 years or more

1. how many hours a week do you work? ________ hours per week*
   *** *if you have a zero-hour contract, please enter the average number of hours you work per week*
2. Have you received training or a course on palliative care for people with ID?

- No
- Yes, namely_____________________________________________________

1. Do you need (more) training or a course on palliative care for people with ID?

- No
- Yes, namely____________________________________________________
